# (2,4,6-Trimethyl­phen­yl){2-[*N*-(2,4,6-trimethyl­phen­yl)formamido]­eth­yl}ammonium chloride

**DOI:** 10.1107/S1600536812044595

**Published:** 2012-11-03

**Authors:** Monisola I. Ikhile, Muhammad D. Bala

**Affiliations:** a School of Chemistry, University of KwaZulu-Natal, Westville Campus, Private Bag X54001, Durban 4000, South Africa

## Abstract

In the title salt, C_21_H_29_N_2_O^+^·Cl^−^, the benzene rings form a dihedral angle of 6.13 (1)°. In the crystal, N—H⋯Cl hydrogen bonds link the cations and anions into chains extending along the *c* axis.

## Related literature

For closely related compounds, see: Kocher & Hermann (1997 ▶); Denk *et al.* (2001 ▶).
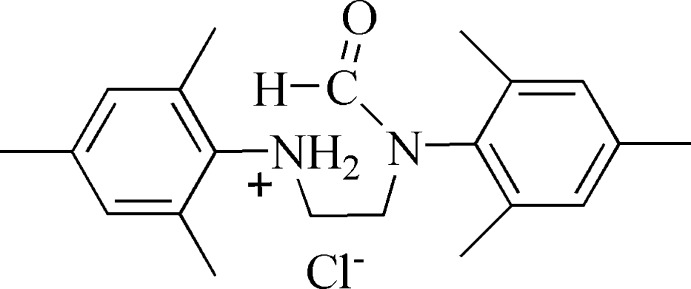


## Experimental

### 

#### Crystal data

C_21_H_29_N_2_O^+^·Cl^−^
*M* *_r_* = 360.91
Triclinic, 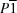

*a* = 8.2516 (2) Å
*b* = 8.8822 (2) Å
*c* = 14.7524 (4) Å
α = 74.857 (2)°
β = 86.315 (2)°
γ = 74.635 (2)°
*V* = 1006.38 (5) Å^3^
*Z* = 2
Mo *K*α radiation
μ = 0.20 mm^−1^
*T* = 173 K
0.36 × 0.21 × 0.11 mm

#### Data collection

Bruker APEXII CCD diffractometer
19610 measured reflections
4853 independent reflections
2975 reflections with *I* > 2σ(*I*)
*R*_int_ = 0.062

#### Refinement

*R*[*F* ^2^ > 2σ(*F* ^2^)] = 0.044
*wR*(*F* ^2^) = 0.107
*S* = 0.90
4853 reflections
232 parameters
H-atom parameters constrained
Δρ_max_ = 0.31 e Å^−3^
Δρ_min_ = −0.25 e Å^−3^

### 

Data collection: *APEX2* (Bruker, 2005 ▶); cell refinement: *SAINT* (Bruker, 2005 ▶); data reduction: *SAINT*; program(s) used to solve structure: *SHELXTL* (Sheldrick, 2008 ▶); program(s) used to refine structure: *SHELXTL*; molecular graphics: *SHELXTL*; software used to prepare material for publication: *SHELXTL*.

## Supplementary Material

Crystal structure: contains datablock(s) I, global. DOI: 10.1107/S1600536812044595/xu5637sup1.cif

Structure factors: contains datablock(s) I. DOI: 10.1107/S1600536812044595/xu5637Isup2.hkl

Additional supplementary materials:  crystallographic information; 3D view; checkCIF report

## Figures and Tables

**Table 1:** Hydrogen-bond geometry (Å, °)

*D*—H⋯*A*	*D*—H	H⋯*A*	*D*⋯*A*	*D*—H⋯*A*
N2—H2*A*⋯Cl1	0.92	2.23	3.0585 (14)	149
N2—H2*B*⋯Cl1^i^	0.92	2.17	3.0531 (14)	161

Symmetry code: (i) 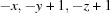.

**Fig. 1.**
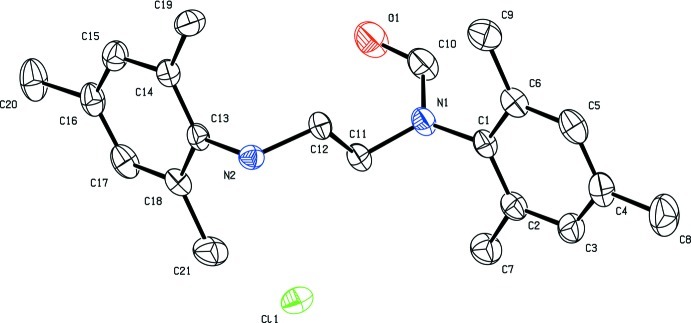
ORTEP diagram of compound (I). Thermal ellipsoids are represented at the 50% probability level. Hydrogen atoms are eliminated for clarity.

**Fig. 2.**
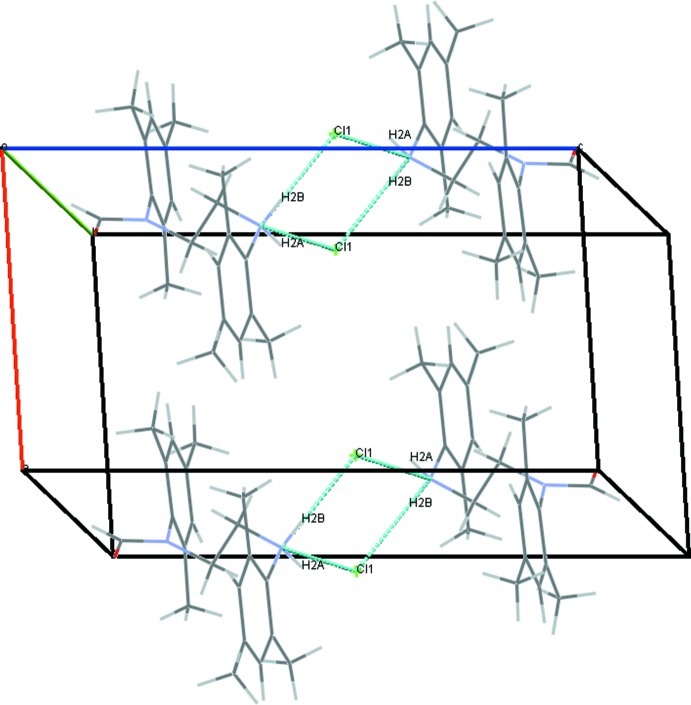
Packing in the title compound showing the N—H···Cl hydrogen bonds.

## supplementary crystallographic information

### Comment

The title compound was isolated from the hydrolysis of an imidazolium based salt in a ring opening reaction. Stable *N*-heterocyclic carbene ligands are often prepared from imidazolium based salt precursors. However the reaction must be conducted under strict anaerobic conditions using Schlenk techniques, otherwise attack by moisture will lead to the isolation of products such as (I).

In (I) (Fig.1), the planes of the two phenyl rings (mean: C1–C6 and mean: C13–C18) form a dihedral angle of 6.13 (1)°. In the crystal, intermolecular N—H···Cl hydrogen bonds (Table 1) link the molecules into chains extending along the crystallographic *c* axis with a separation of 2.17–2.23 Å (Fig. 2).

### Experimental

A mixture of 1,3–bis(2,4,6-trimethyl phenyl)imidazolinium chloride (0.101 g, 0.29 mmol) and potassium *tert*-butoxide (0.039 g, 0.35 mmol) were dissolved in 20 ml of tetrahydrofuran and stirred at room temperature for 30 min. After evaporating the solvent, the free carbene was extracted in warm toluene (2 *x* 20 ml). FeCl_2_ (0.037 g, 0.29 mmol) was added to the toluene solution and refluxed for 24 h at 90 °C. Removal of the solvent gave a residue that was purified by recrystallization from CH_2_Cl_2_/hexane to yield orange crystals of (I). Yield: 0.05 g, 51%; m.p. 180 °C.

### Refinement

H-atoms were refined using a riding model, with C—H = 0.95 Å and *U*_iso_(H) = 1.2*U*_eq_(C) for aromatic and C—H = 0.98 Å and *U*_iso_(H) = 1.5*U*_eq_(C) for CH_3_. The amino H atoms were placed in calculated positions with N—H = 0.92 Å and refined in a riding mode with *U*_iso_(H) = 1.2*U*_eq_(N).

### Crystal data

**Table:**

C_21_H_29_N_2_O^+^·Cl^−^	*Z* = 2
*M**_r_* = 360.91	*F*(000) = 388
Triclinic, *P*1	*D*_x_ = 1.191 Mg m^−^^3^
Hall symbol: -P 1	Mo *K*α radiation, λ = 0.71073 Å
*a* = 8.2516 (2) Å	Cell parameters from 3132 reflections
*b* = 8.8822 (2) Å	θ = 2.5–24.0°
*c* = 14.7524 (4) Å	µ = 0.20 mm^−^^1^
α = 74.857 (2)°	*T* = 173 K
β = 86.315 (2)°	Block, colourless
γ = 74.635 (2)°	0.36 × 0.21 × 0.11 mm
*V* = 1006.38 (5) Å^3^	

### Data collection

**Table:**

Bruker APEXII CCD diffractometer	2975 reflections with *I* > 2σ(*I*)
Radiation source: fine-focus sealed tube	*R*_int_ = 0.062
Graphite monochromator	θ_max_ = 28.0°, θ_min_ = 1.4°
φ and ω scans	*h* = −10→10
19610 measured reflections	*k* = −11→11
4853 independent reflections	*l* = −19→19

### Refinement

**Table:**

Refinement on *F*^2^	Primary atom site location: structure-invariant direct methods
Least-squares matrix: full	Secondary atom site location: difference Fourier map
*R*[*F*^2^ > 2σ(*F*^2^)] = 0.044	Hydrogen site location: inferred from neighbouring sites
*wR*(*F*^2^) = 0.107	H-atom parameters constrained
*S* = 0.90	*w* = 1/[σ^2^(*F*_o_^2^) + (0.0478*P*)^2^] where *P* = (*F*_o_^2^ + 2*F*_c_^2^)/3
4853 reflections	(Δ/σ)_max_ = 0.001
232 parameters	Δρ_max_ = 0.31 e Å^−^^3^
0 restraints	Δρ_min_ = −0.25 e Å^−^^3^

### Special details

**Table:**

Experimental. IR (ATR cm^-1^): 2918, 2718, 2607, 1669, 1568, 1482, 1446, 1381, 1345, 1306, 1285, 1207, 1193, 1033, 850, 781, 705, 577, 477, 425; δ_H_ (400 MHz, CDCl_3_): 1.60 (6*H*, s, CH_3_), 2.16 (12*H*, m, CH_3_), 2.58 (3*H*, s, NH_2_), 3.76 (2*H*, s, NCH_2_NCHO), 6.83 (3*H*, s, ArH), 6.88 (2*H*, s, ArH) and 7.89 p.p.m. (1*H*, s, NCHO); δ_C_ (100 MHz, CDCl_3_): 18.09 (C_6_H_2_(CH_3_)_3_), 19.35 (C_6_H_2_(CH_3_)_3_), 21.15 (C_6_H_2_(CH_3_)_3_), 23.17 (C_6_H_2_(CH_3_)_3_), 51.12 (NHCH_2_), 52.08 (NCH_2_), 128.27, 130.18, 134.72, 135.91, 138.54, 140.21, 145.23 and 160.28 p.p.m. (NCHO); HRMS (ESI), Found, 325.22737 (*M*^+^ - Cl^-^) Calculated for C_21_H_29_N_2_O 325.22799 (*M*^+^ - Cl^-^).
Geometry. All e.s.d.'s (except the e.s.d. in the dihedral angle between two l.s. planes) are estimated using the full covariance matrix. The cell e.s.d.'s are taken into account individually in the estimation of e.s.d.'s in distances, angles and torsion angles; correlations between e.s.d.'s in cell parameters are only used when they are defined by crystal symmetry. An approximate (isotropic) treatment of cell e.s.d.'s is used for estimating e.s.d.'s involving l.s. planes.
Refinement. Refinement of *F*^2^ against ALL reflections. The weighted *R*-factor *wR* and goodness of fit *S* are based on *F*^2^, conventional *R*-factors *R* are based on *F*, with *F* set to zero for negative *F*^2^. The threshold expression of *F*^2^ > σ(*F*^2^) is used only for calculating *R*-factors(gt) *etc*. and is not relevant to the choice of reflections for refinement. *R*-factors based on *F*^2^ are statistically about twice as large as those based on *F*, and *R*- factors based on ALL data will be even larger.

### Fractional atomic coordinates and isotropic or equivalent isotropic displacement parameters (Å^2^)

**Table:**

	*x*	*y*	*z*	*U*_iso_*/*U*_eq_	
C1	−0.0762 (2)	0.8248 (2)	0.13320 (12)	0.0263 (4)	
C2	−0.0175 (2)	0.9590 (2)	0.13117 (12)	0.0298 (4)	
C3	−0.1346 (2)	1.1060 (2)	0.12548 (13)	0.0347 (5)	
H3	−0.0958	1.1971	0.1266	0.042*	
C4	−0.3055 (2)	1.1251 (2)	0.11826 (13)	0.0365 (5)	
C5	−0.3594 (2)	0.9905 (2)	0.11714 (13)	0.0373 (5)	
H5	−0.4761	1.0021	0.1104	0.045*	
C6	−0.2486 (2)	0.8385 (2)	0.12561 (12)	0.0318 (4)	
C7	0.1675 (2)	0.9497 (2)	0.13254 (14)	0.0390 (5)	
H7A	0.2107	0.9029	0.1969	0.058*	
H7B	0.1844	1.0584	0.1096	0.058*	
H7C	0.2277	0.8817	0.0920	0.058*	
C8	−0.4291 (3)	1.2871 (3)	0.11207 (17)	0.0573 (6)	
H8A	−0.4089	1.3296	0.1641	0.086*	
H8B	−0.5439	1.2744	0.1159	0.086*	
H8C	−0.4145	1.3622	0.0522	0.086*	
C9	−0.3162 (2)	0.6959 (2)	0.12768 (15)	0.0441 (5)	
H9A	−0.2934	0.6669	0.0676	0.066*	
H9B	−0.4378	0.7239	0.1383	0.066*	
H9C	−0.2616	0.6041	0.1786	0.066*	
C10	0.0609 (2)	0.5899 (2)	0.07631 (14)	0.0392 (5)	
H10	0.0013	0.6435	0.0190	0.047*	
C11	0.1348 (2)	0.5881 (2)	0.23205 (12)	0.0288 (4)	
H11A	0.1508	0.6696	0.2630	0.035*	
H11B	0.2471	0.5234	0.2188	0.035*	
C12	0.0402 (2)	0.4789 (2)	0.29708 (12)	0.0277 (4)	
H12A	−0.0680	0.5452	0.3143	0.033*	
H12B	0.0153	0.4042	0.2638	0.033*	
C13	0.2789 (2)	0.2444 (2)	0.37526 (12)	0.0249 (4)	
C14	0.2386 (2)	0.1044 (2)	0.36930 (12)	0.0265 (4)	
C15	0.3711 (2)	−0.0292 (2)	0.36709 (12)	0.0320 (4)	
H15	0.3467	−0.1262	0.3634	0.038*	
C16	0.5376 (2)	−0.0257 (2)	0.37002 (12)	0.0328 (4)	
C17	0.5706 (2)	0.1165 (2)	0.37419 (13)	0.0345 (5)	
H17	0.6843	0.1205	0.3750	0.041*	
C18	0.4440 (2)	0.2546 (2)	0.37721 (12)	0.0280 (4)	
C19	0.0599 (2)	0.0917 (2)	0.36649 (14)	0.0345 (5)	
H19A	−0.0098	0.1503	0.4090	0.052*	
H19B	0.0581	−0.0221	0.3863	0.052*	
H19C	0.0155	0.1386	0.3024	0.052*	
C20	0.6787 (2)	−0.1744 (2)	0.36982 (15)	0.0471 (6)	
H20A	0.6388	−0.2712	0.3968	0.071*	
H20B	0.7730	−0.1756	0.4074	0.071*	
H20C	0.7157	−0.1727	0.3052	0.071*	
C21	0.4916 (2)	0.4051 (2)	0.38237 (15)	0.0409 (5)	
H21A	0.6129	0.3795	0.3934	0.061*	
H21B	0.4309	0.4457	0.4340	0.061*	
H21C	0.4620	0.4877	0.3231	0.061*	
N1	0.04243 (17)	0.66963 (18)	0.14387 (10)	0.0289 (3)	
N2	0.13837 (16)	0.38311 (16)	0.38442 (10)	0.0246 (3)	
H2A	0.1811	0.4518	0.4072	0.030*	
H2B	0.0652	0.3452	0.4287	0.030*	
O1	0.14983 (17)	0.45239 (18)	0.08378 (10)	0.0544 (4)	
Cl1	0.13717 (5)	0.66160 (5)	0.47043 (3)	0.03312 (14)	

### Atomic displacement parameters (Å^2^)

**Table:**

	*U*^11^	*U*^22^	*U*^33^	*U*^12^	*U*^13^	*U*^23^
C1	0.0249 (9)	0.0308 (10)	0.0200 (9)	−0.0033 (8)	−0.0015 (7)	−0.0046 (8)
C2	0.0297 (10)	0.0356 (11)	0.0228 (9)	−0.0091 (9)	−0.0013 (8)	−0.0041 (8)
C3	0.0427 (11)	0.0315 (11)	0.0277 (10)	−0.0080 (9)	−0.0031 (9)	−0.0043 (9)
C4	0.0357 (11)	0.0368 (12)	0.0290 (10)	0.0015 (9)	−0.0019 (9)	−0.0052 (9)
C5	0.0236 (9)	0.0494 (13)	0.0331 (11)	−0.0026 (9)	−0.0023 (8)	−0.0069 (10)
C6	0.0280 (10)	0.0399 (12)	0.0261 (10)	−0.0075 (9)	−0.0019 (8)	−0.0065 (9)
C7	0.0321 (10)	0.0436 (12)	0.0413 (12)	−0.0132 (9)	−0.0015 (9)	−0.0071 (10)
C8	0.0571 (14)	0.0446 (14)	0.0557 (15)	0.0123 (11)	−0.0079 (12)	−0.0117 (12)
C9	0.0331 (11)	0.0495 (13)	0.0516 (14)	−0.0140 (10)	−0.0051 (10)	−0.0115 (11)
C10	0.0345 (11)	0.0473 (13)	0.0331 (11)	0.0000 (10)	−0.0053 (9)	−0.0152 (10)
C11	0.0262 (9)	0.0323 (10)	0.0252 (9)	−0.0033 (8)	−0.0053 (7)	−0.0056 (8)
C12	0.0225 (9)	0.0274 (10)	0.0295 (10)	−0.0014 (8)	−0.0067 (7)	−0.0044 (8)
C13	0.0222 (9)	0.0263 (10)	0.0226 (9)	0.0004 (7)	−0.0028 (7)	−0.0058 (8)
C14	0.0265 (9)	0.0268 (10)	0.0232 (9)	−0.0025 (8)	−0.0016 (7)	−0.0052 (8)
C15	0.0372 (11)	0.0276 (10)	0.0275 (10)	−0.0028 (8)	0.0009 (8)	−0.0067 (8)
C16	0.0313 (10)	0.0345 (11)	0.0242 (10)	0.0044 (9)	0.0000 (8)	−0.0060 (8)
C17	0.0208 (9)	0.0479 (12)	0.0291 (10)	−0.0014 (9)	0.0001 (8)	−0.0077 (9)
C18	0.0232 (9)	0.0363 (11)	0.0234 (9)	−0.0056 (8)	−0.0026 (7)	−0.0074 (8)
C19	0.0322 (10)	0.0308 (11)	0.0443 (12)	−0.0095 (9)	−0.0002 (9)	−0.0143 (9)
C20	0.0418 (12)	0.0468 (13)	0.0361 (12)	0.0127 (10)	0.0024 (9)	−0.0070 (10)
C21	0.0272 (10)	0.0490 (13)	0.0514 (13)	−0.0133 (9)	−0.0024 (9)	−0.0172 (11)
N1	0.0295 (8)	0.0312 (9)	0.0227 (8)	−0.0015 (7)	−0.0042 (6)	−0.0065 (7)
N2	0.0225 (7)	0.0259 (8)	0.0264 (8)	−0.0065 (6)	0.0002 (6)	−0.0079 (6)
O1	0.0514 (9)	0.0540 (10)	0.0525 (10)	0.0147 (8)	−0.0150 (8)	−0.0295 (8)
Cl1	0.0312 (3)	0.0314 (3)	0.0414 (3)	−0.0118 (2)	0.0066 (2)	−0.0148 (2)

### Geometric parameters (Å, º)

**Table:**

C1—C2	1.394 (2)	C11—H11B	0.9900
C1—C6	1.404 (2)	C12—N2	1.495 (2)
C1—N1	1.440 (2)	C12—H12A	0.9900
C2—C3	1.389 (2)	C12—H12B	0.9900
C2—C7	1.508 (2)	C13—C18	1.392 (2)
C3—C4	1.383 (3)	C13—C14	1.394 (2)
C3—H3	0.9500	C13—N2	1.4802 (19)
C4—C5	1.385 (3)	C14—C15	1.391 (2)
C4—C8	1.511 (3)	C14—C19	1.513 (2)
C5—C6	1.396 (2)	C15—C16	1.386 (2)
C5—H5	0.9500	C15—H15	0.9500
C6—C9	1.506 (3)	C16—C17	1.378 (3)
C7—H7A	0.9800	C16—C20	1.512 (2)
C7—H7B	0.9800	C17—C18	1.393 (2)
C7—H7C	0.9800	C17—H17	0.9500
C8—H8A	0.9800	C18—C21	1.511 (2)
C8—H8B	0.9800	C19—H19A	0.9800
C8—H8C	0.9800	C19—H19B	0.9800
C9—H9A	0.9800	C19—H19C	0.9800
C9—H9B	0.9800	C20—H20A	0.9800
C9—H9C	0.9800	C20—H20B	0.9800
C10—O1	1.227 (2)	C20—H20C	0.9800
C10—N1	1.345 (2)	C21—H21A	0.9800
C10—H10	0.9500	C21—H21B	0.9800
C11—N1	1.463 (2)	C21—H21C	0.9800
C11—C12	1.513 (2)	N2—H2A	0.9200
C11—H11A	0.9900	N2—H2B	0.9200
			
C2—C1—C6	121.13 (16)	N2—C12—H12B	109.2
C2—C1—N1	119.19 (15)	C11—C12—H12B	109.2
C6—C1—N1	119.69 (15)	H12A—C12—H12B	107.9
C3—C2—C1	118.23 (16)	C18—C13—C14	122.59 (15)
C3—C2—C7	119.68 (16)	C18—C13—N2	119.86 (15)
C1—C2—C7	122.08 (16)	C14—C13—N2	117.49 (14)
C4—C3—C2	122.63 (18)	C15—C14—C13	117.36 (15)
C4—C3—H3	118.7	C15—C14—C19	119.38 (16)
C2—C3—H3	118.7	C13—C14—C19	123.25 (15)
C3—C4—C5	117.69 (17)	C16—C15—C14	122.21 (17)
C3—C4—C8	121.08 (19)	C16—C15—H15	118.9
C5—C4—C8	121.24 (18)	C14—C15—H15	118.9
C4—C5—C6	122.44 (17)	C17—C16—C15	118.06 (16)
C4—C5—H5	118.8	C17—C16—C20	121.05 (17)
C6—C5—H5	118.8	C15—C16—C20	120.88 (18)
C5—C6—C1	117.82 (17)	C16—C17—C18	122.75 (17)
C5—C6—C9	119.69 (16)	C16—C17—H17	118.6
C1—C6—C9	122.49 (17)	C18—C17—H17	118.6
C2—C7—H7A	109.5	C13—C18—C17	117.00 (16)
C2—C7—H7B	109.5	C13—C18—C21	123.79 (16)
H7A—C7—H7B	109.5	C17—C18—C21	119.21 (16)
C2—C7—H7C	109.5	C14—C19—H19A	109.5
H7A—C7—H7C	109.5	C14—C19—H19B	109.5
H7B—C7—H7C	109.5	H19A—C19—H19B	109.5
C4—C8—H8A	109.5	C14—C19—H19C	109.5
C4—C8—H8B	109.5	H19A—C19—H19C	109.5
H8A—C8—H8B	109.5	H19B—C19—H19C	109.5
C4—C8—H8C	109.5	C16—C20—H20A	109.5
H8A—C8—H8C	109.5	C16—C20—H20B	109.5
H8B—C8—H8C	109.5	H20A—C20—H20B	109.5
C6—C9—H9A	109.5	C16—C20—H20C	109.5
C6—C9—H9B	109.5	H20A—C20—H20C	109.5
H9A—C9—H9B	109.5	H20B—C20—H20C	109.5
C6—C9—H9C	109.5	C18—C21—H21A	109.5
H9A—C9—H9C	109.5	C18—C21—H21B	109.5
H9B—C9—H9C	109.5	H21A—C21—H21B	109.5
O1—C10—N1	124.35 (18)	C18—C21—H21C	109.5
O1—C10—H10	117.8	H21A—C21—H21C	109.5
N1—C10—H10	117.8	H21B—C21—H21C	109.5
N1—C11—C12	110.66 (14)	C10—N1—C1	121.01 (15)
N1—C11—H11A	109.5	C10—N1—C11	118.12 (15)
C12—C11—H11A	109.5	C1—N1—C11	120.64 (14)
N1—C11—H11B	109.5	C13—N2—C12	116.63 (13)
C12—C11—H11B	109.5	C13—N2—H2A	108.1
H11A—C11—H11B	108.1	C12—N2—H2A	108.1
N2—C12—C11	111.89 (13)	C13—N2—H2B	108.1
N2—C12—H12A	109.2	C12—N2—H2B	108.1
C11—C12—H12A	109.2	H2A—N2—H2B	107.3
			
C6—C1—C2—C3	2.7 (3)	C19—C14—C15—C16	−179.61 (17)
N1—C1—C2—C3	−176.89 (16)	C14—C15—C16—C17	−0.8 (3)
C6—C1—C2—C7	−175.73 (17)	C14—C15—C16—C20	178.54 (17)
N1—C1—C2—C7	4.6 (3)	C15—C16—C17—C18	1.2 (3)
C1—C2—C3—C4	−2.5 (3)	C20—C16—C17—C18	−178.12 (17)
C7—C2—C3—C4	176.02 (17)	C14—C13—C18—C17	−0.8 (3)
C2—C3—C4—C5	0.3 (3)	N2—C13—C18—C17	176.08 (15)
C2—C3—C4—C8	−179.90 (18)	C14—C13—C18—C21	179.17 (17)
C3—C4—C5—C6	1.8 (3)	N2—C13—C18—C21	−3.9 (3)
C8—C4—C5—C6	−178.02 (19)	C16—C17—C18—C13	−0.4 (3)
C4—C5—C6—C1	−1.6 (3)	C16—C17—C18—C21	179.60 (17)
C4—C5—C6—C9	177.59 (18)	O1—C10—N1—C1	−174.15 (17)
C2—C1—C6—C5	−0.8 (3)	O1—C10—N1—C11	0.4 (3)
N1—C1—C6—C5	178.83 (15)	C2—C1—N1—C10	−117.52 (19)
C2—C1—C6—C9	−179.92 (17)	C6—C1—N1—C10	62.9 (2)
N1—C1—C6—C9	−0.3 (3)	C2—C1—N1—C11	68.1 (2)
N1—C11—C12—N2	175.09 (13)	C6—C1—N1—C11	−111.52 (18)
C18—C13—C14—C15	1.2 (3)	C12—C11—N1—C10	−83.4 (2)
N2—C13—C14—C15	−175.77 (15)	C12—C11—N1—C1	91.09 (18)
C18—C13—C14—C19	−179.60 (17)	C18—C13—N2—C12	106.18 (18)
N2—C13—C14—C19	3.4 (2)	C14—C13—N2—C12	−76.74 (19)
C13—C14—C15—C16	−0.4 (3)	C11—C12—N2—C13	−75.89 (18)

### Hydrogen-bond geometry (Å, º)

**Table:**

*D*—H···*A*	*D*—H	H···*A*	*D*···*A*	*D*—H···*A*
N2—H2*A*···Cl1	0.92	2.23	3.0585 (14)	149
N2—H2*B*···Cl1^i^	0.92	2.17	3.0531 (14)	161

Symmetry code: (i) −*x*, −*y*+1, −*z*+1.
